# Cardiovascular Disease in Adult Cancer Survivors: a Review of Current Evidence, Strategies for Prevention and Management, and Future Directions for Cardio-oncology

**DOI:** 10.1007/s11912-022-01309-w

**Published:** 2022-07-07

**Authors:** Jaidyn Muhandiramge, John R. Zalcberg, G. J. van Londen, Erica T. Warner, Prudence R. Carr, Andrew Haydon, Suzanne G. Orchard

**Affiliations:** 1 Department of Epidemiology and Preventive Medicine, School of Public Health and Preventive Medicine, Monash University, 553 St Kilda Road, Melbourne, VIC 3004 Australia; 2 Austin Health, Heidelberg, VIC Australia; 3 Department of Medical Oncology, Alfred Hospital, Melbourne, VIC Australia; 4 Department of Medicine, University of Pittsburgh, Pittsburgh, PA USA; 5 Clinical and Translational Epidemiology Unit, MGH Cancer Center, Massachusetts General Hospital and Harvard Medical School, Boston, MA USA

**Keywords:** Cancer, Cardiovascular disease, Myocardial infarction, Heart failure, Stroke, Aging

## Abstract

**Purpose of Review:**

Cardiovascular disease is long-term complication of both cancer and anti-cancer treatment and can have significant ramifications for health-related quality of life and mortality. This narrative review explores the current evidence linking cardiovascular disease and cancer, as well as exploring strategies for the prevention and management of cardiovascular disease, and outlines future opportunities in the field of cardio-oncology.

**Recent Findings:**

Cancer confers risk for various cardiovascular diseases including heart failure, cardiomyopathy, arrhythmia, coronary heart disease, stroke, venous thromboembolism, and valvular heart disease. Cancer treatment, in particular agents such as platinum-based chemotherapy, anthracyclines, hormonal treatments, and thoracic radiotherapy, further increases risk. While cardiovascular disease can be identified early and effectively managed in cancer survivors, cardiovascular screening and management does not typically feature in routine long-term cancer care of adult cancer survivors.

**Summary:**

Cancer and cancer treatment can accelerate the development of cardiovascular disease. Further research into screening and management strategies for cardiovascular disease, along with evidence-based guidelines, is required to ensure adult cancer survivors receive appropriate long-term care.

**Supplementary Information:**

The online version contains supplementary material available at 10.1007/s11912-022-01309-w.

## Introduction

Both cancer and aging are inextricably linked by a central mechanism: an accumulation of cellular damage over time resulting in progressive cellular dysfunction and ensuing deficits across various organ systems [1, 2]. Aging is therefore widely accepted to be a strong risk factor for cancer [3]. However, aging processes may also be accelerated as a result of cancer and cancer treatment [4•, 5]. The functional outcomes of aging, sometimes referred to as clinical manifestations of aging, include various deficits such as functional decline, frailty, cognitive impairment, and chronic disease [4•, 5]. One important clinical manifestation of aging not often linked with cancer is cardiovascular disease (CVD) [6], a common group of disorders that typically encompass cardiac disease, arterial disease, and cerebrovascular disease (i.e. stroke) [7]. Many of these aging outcomes, however, are relatively under-represented in research.

In 2018, the National Cancer Institute assembled a think tank titled “Measuring Aging and Identifying Aging Phenotypes in Cancer Survivors” to explore the challenges that exist in measuring, and subsequently preventing accelerated aging in cancer survivors. The resulting report identified a need for further research surrounding the clinical manifestations of aging in this cohort [4•]. Of all the clinical manifestations of aging, CVD is one that can be identified early, effectively managed, and in many cases prevented [8]. In spite of this, CVD screening and management does not typically feature in routine long-term cancer care. The intersection of CVD and cancer, along with a newfound understanding of the importance of cardiac care to patients with cancer, has led to the development of a relatively new field: cardio-oncology.

This narrative review explores the current evidence surrounding the increased risk of CVD due to cancer, as well as strategies for its prevention and management, and outlines where future research in cardio-oncology might be directed. The search strategy for this review can be found in the Supplementary Material.

## Impact of Cancer on Risk of Cardiovascular Disease

Several large-scale studies support a positive association between CVD risk and cancer across multiple cancer types. Some of the literature on this topic is outlined below, with a preference for landmark or recent studies, large sample sizes, systematic reviews or meta-analyses, adjustment for anti-cancer treatment, and comparison with cancer-free controls.

Two analyses by Zoller et al. [9••, 10••] using large cohorts provide some of the earlier evidence for this link. In the first analysis, Zoller et al. [10••] investigated 820,491 Swedish individuals with cancer and compared them with the entire cancer-free population of Sweden. They indicated that while coronary heart disease risk was highest in the 6 months immediately post-diagnosis (Standardized Incidence Ratio [SIR] 1.70, 95% CI 1.66–1.75) and did decrease over time, it nonetheless remained elevated even after 10 years following a cancer diagnosis (SIR 1.07, 95% CI 1.04–1.11) [10••]. Zoller et al.’s [9••] analysis of the same cohort demonstrated over double the risk of stroke during the first 6 months following a diagnosis of cancer (SIR 2.2, 95% CI 2.0–2.3), with the risk attenuating rapidly, but nonetheless remaining elevated for 10 years post-diagnosis (SIR 1.2, 95% CI 1.1–1.3). Notably, neither analysis had access to cardiovascular risk factor data (e.g. smoking, weight, diet) nor cancer treatment data and therefore could not adjust for these variables, although they did adjust for basic demographic factors such as age and gender, along with relevant comorbidities such as hypertension and diabetes. Both studies also used hospitalisation for coronary heart disease or stroke as events, thereby excluding more minor CVD events treated in an outpatient setting.

More recently, a UK-based study by Strongman et al. [11••] conducted a time-to-event analysis of 108,215 patients with cancer and demonstrated persistently increased risks of heart failure, cardiomyopathy, arrhythmia, coronary heart disease, stroke, and valvular heart disease following the diagnosis of various cancers. While the risk of some events such as venous thromboembolism reduced over time, others such as heart failure and cardiomyopathy continued to increase over a period of over 12 years [11••]. Schoormans et al.’s [12] 2018 analysis of 32,757 Dutch cancer survivors corroborated these findings, with survivors of prostate, lung, and tracheal cancer demonstrating an increased risk of incident CVD over a follow-up period of up to 13 years. Notably, CVD risk in the latter two cancer types remained statistically significant after adjusting for cancer treatment type and CVD risk factors [12]. Paterson et al. [13••] similarly demonstrated statistically significant increases in the risk of stroke (HR 1.44, 95% CI 1.41–1.47), heart failure (HR 1.62, 95% CI 1.59–1.65), and pulmonary embolism (HR 3.43, 95% CI 3.37–3.50) in patients with cancer. The risk was further increased in patients with genitourinary, gastrointestinal, thoracic, nervous system, and hematologic malignancies. The study had several methodological strengths including a large sample size (*n*^total^ = 4,519,243; *n*^cancer^ = 224,016), long follow-up time (11.8 years), and adjustment for 31 comorbidities including nine cardiovascular comorbidities [13••]. A recent 2021 meta-analysis confirmed increased stroke risk in an analysis of 10,479,530 cancer survivors across 21 cohort studies, demonstrating a relative risk of 1.66 (95% CI 1.35–2.04) when compared to cancer-free controls. A diagnosis of head and neck, blood, lung, pancreas, and stomach cancer in particular was associated with a consistently significant increase in risk [14••].

## Mechanisms Driving Cancer-Related Cardiovascular Disease

A causal association between the diagnosis (and/or treatment) of a tumour itself and CVD may exist, with most theories implicating cancer-induced inflammation as the common link [15]. There is evidence to suggest that activation of certain oncogenes and inactivation of tumour-suppressor genes may produce pro-inflammatory molecules, creating an inflammatory micro-environment that underpins the subsequent development of CVD [16]. C-reactive protein is one acute phase reactant that can be raised as a result of this inflammatory response and can be used as a predictor of progressive disease in some cancer types [17]. An increased incidence of CVD seen in patients with metastatic disease may support this link [9••], given the association between a greater total tumour volume and increased inflammation. However, it should be noted that patients with advanced disease are also more likely to receive greater amounts of treatment and may therefore be impacted by this rather than the cancer itself. Nevertheless, it is clear that tumours can promote the release of pro-inflammatory cytokines and atherosclerotic plaque formation [18, 19], both mechanisms that underly the development of heart disease [6]. Early evidence also exists supporting a link between clonal hematopoiesis, cancer, and CVD. Clonal hematopoiesis is a condition characterized by the proliferation of hematopoietic stem cells carrying certain somatic mutations, and is typically associated with an increased risk of hematologic malignancy [20]. More recently, clonal hematopoiesis has also been linked to CVD, likely as a result of inflammatory processes accelerating atherosclerosis and venous thrombosis [21]. In a nested case–control of 8255 individuals, Jaiswal et al. demonstrated a 1.9-fold increase in the risk of coronary heart disease in carriers of clonal hematopoiesis compared with non-carriers [22]. Clonal hematopoiesis may therefore represent a shared inflammation-mediated mechanism by which patients with cancer develop CVD.

Stroke in cancer survivors is a more complicated phenomenon and may relate to several factors including direct tumour effects (e.g. vascular invasion, cancer-associated inflammation, tumour emboli) and coagulopathy [23]. Coagulopathy appears to be the primary driving factor in cancer-related stroke and is in turn driven by various mechanisms including thrombocytosis, tumour-expressed procoagulants, inflammation, and disseminated intravascular coagulopathy [23]. As is the case for heart disease, evidence suggests that cancer aggressiveness may influence stroke risk [9, 24], again suggesting a link between tumour load and cancer-associated inflammation.

**Fig. 1:**
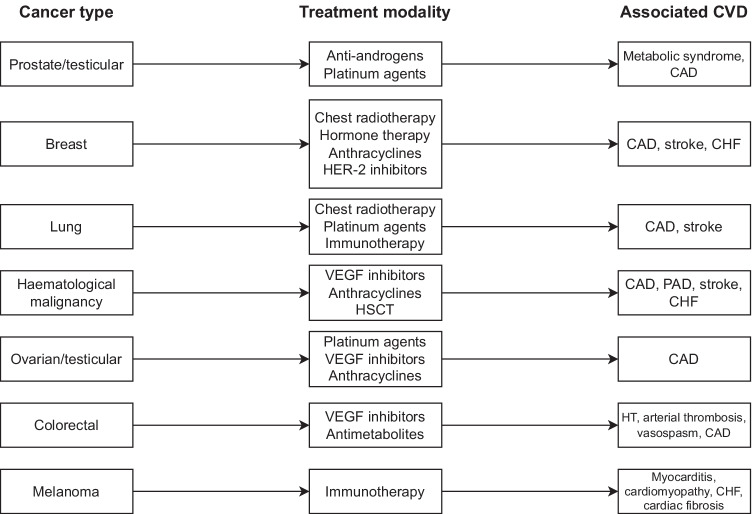
Treatment modalities commonly associated with cardiovascular disease, adapted from Kolominsky et al. [79] Abbreviations used: CAD = coronary artery disease, PAD = peripheral arterial disease, HT = hypertension, HSCT = Hematopoietic stem-cell transplantation, CHF = congestive heart failure

Anti-cancer treatment appears to have an additional direct impact on heart disease. Radiotherapy has been shown to substantially increase cardiovascular risk in patients with cancers requiring thoracic radiation such as breast cancer, lung cancer, and Hodgkin’s lymphoma [25–27]. Notably, this risk is proportional to the proximity of the tumour site receiving radiation to the heart and, therefore, the total cardiac radiation dose received [28]. As a result, there is little evidence to suggest irradiation of tumours distant to the thorax (e.g. pelvic irradiation for prostate cancer) can cause similar levels of cardiac damage. Several systemic therapies including cytotoxic chemotherapy (e.g. anthracyclines), hormonal therapy (e.g. selective oestrogen receptor modulators, aromatase inhibitors, LHRH agonists and antagonists, androgen receptor antagonists) [29], targeted therapy (e.g. trastuzumab, kinase inhibitors), and immunotherapy (e.g. ipilimumab) have also been linked to CVD [30, 31]. Hormone-based treatments in particular confer risk for various cardiovascular diseases including dyslipidaemia, hypertension, venous thromboembolism, stroke, arrhythmia, myocardial infarction, and heart failure. Notably, the effects of each individual agent vary significantly due to their heterogeneous mechanisms of action, and while some increase risk for certain cardiovascular diseases, others provide a protective benefit [29]. The literature similarly supports a link between cancer treatment and cerebrovascular disease. In a pooled meta-analysis of 12 studies, Huang et al. [32] reported that patients who had received radiotherapy (to any site) experienced stroke at over double the rate of patients with cancer who had not received radiotherapy. Similarly, chemotherapy has been reported to increase stroke risk through a number of mechanisms, including endothelial dysfunction and disruption of normal coagulation and haemostasis [33]. Some cytotoxic chemotherapeutic agents, particularly platinum-based compounds such as cisplatin, confer considerable risk for stroke [33]. The mechanism driving this is less clear but may involve the release of prothrombotic endothelial and platelet-derived microparticles seen following cisplatin infusion [34]. Conversely, targeted therapy with anti-angiogenic tyrosine kinase inhibitors has not been directly linked to stroke. Some agents such as sunitinib, however, are known to cause thrombocytopenia and hypertension (a class effect common to many of the tyrosine kinase inhibitors), providing a potential link to hemorrhagic stroke [35]. Allogeneic hematopoietic stem-cell transplantation may also contribute to late cardiovascular effects in patients with hematologic malignancy. Tichelli et al. [36] demonstrated increased risk of arterial events in patients who had undergone such transplantation, with this risk persisting for several decades post-transplantation. Many of the cardiac effects of anti-cancer treatments are thought to contribute to the longer term CVD risk seen in cancer survivors, with mediastinal radiation and cardiotoxic chemotherapeutic agents being the most commonly cited culprits [37]. Fig. 1 provides a summary of some of the treatment modalities commonly associated with CVD.

The indirect impact of cancer and cancer treatment on CVD risk should also be considered. Exercise intolerance and subsequent sedentarism is one such sequelae of cancer that is common in survivors and can predispose to CVD [38, 39]. Sedentarism is undoubtedly an important consideration in assessing cardiovascular risk, with a 2016 American Heart Association review suggesting a link between sedentary behaviour and not only CVD risk, but also cardiovascular morbidity and mortality [39]. Sedentarism and a lack of cardiorespiratory fitness can be driven by several cancer-related mechanisms including inactivity secondary to cancer or treatment-related symptoms such as fatigue, pain, or physical limitations [40]. Cancer treatment can have an even more damaging effect on exercise tolerance, particularly in patients treated with pulmonary resection [41], thoracic radiation [42], cardiotoxic systemic agents [30, 31], and hormone therapy (both due to toxicity-related effects such as weakness and muscle wasting [43] and a potentially direct impact on cardiac function [44, 45]).

Another indirect impact of cancer lies in the need for various supportive medications in patients with cancer. Many individuals in this cohort are subject to polypharmacy irrespective of their specific anti-cancer regimen; these include the use of medications such as analgesics, aperients, and anti-emetics [46]. A large number of medications may preclude patients from receiving cardioprotective agents (e.g. beta blockers, angiotensin-converting enzyme inhibitors) due to drug-drug interactions, or clinician hesitance to add to an already significant pill burden. Some of these supportive drugs may also increase CVD risk independently. Steroids, for example, are commonly used in patients with cancer for the management of cancer-related complications such as nausea, and to reduce peritumoral oedema and inflammation [47]. Corticosteroids are well known to cause hypertension, hyperglycemia (often inducing diabetic or pre-diabetic states), dyslipidaemia, and obesity, especially in the high doses and long durations used in cancer, significantly increasing CVD risk [48].

The impact of shared etiological risk factors should also be acknowledged given that several modifiable risk factors can contribute to both cancer and CVD [49]. Smoking, physical inactivity, alcohol consumption, and poor nutrition are some of the most significant modifiable risk factors applicable to both conditions. It is estimated that elimination of these factors could help to prevent over 80% of heart disease, stroke, and type 2 diabetes, as well as 40% of malignancies [49]. Given these shared risk factors, the incidence of CVD in cancer survivors may be, at least in part, attributable to a patient’s lifestyle.

## Clinical Implications of Cancer-Related Cardiovascular Disease

The clinical utility of investigating and subsequently treating CVD in cancer survivors lies in its impact on quality of life and mortality. This impact is aptly summarized by Scott et al. [50], who found that the predicted heart age of male and female cancer survivors was 8.5 and 6.5 years older, respectively, than their actual age. This calculation of heart age, derived from a 10-year cardiovascular disease risk estimate using the Framingham Risk Score, provides a simple picture of the implications of cancer-related CVD.

CVD, and chronic illness in general, is well known to be associated with impaired health-related quality of life in the general population. While there is a paucity of literature investigating long-term health-related quality of life and CVD in cancer cohorts, there is some evidence to suggest impairment in some domains of health-related quality of life in cancer survivors is due to comorbid CVD [51]. Given that the majority of patients with cancer place at least equal weight on quality of life versus length of life [52], it is doubly important to identify and proactively manage CVD in these patients.

The link between CVD and mortality in cancer survivors is somewhat clearer. A 2020 analysis of over seven million patients with cancer revealed that CVD-related mortality is 2.24 times that of the general population (95% CI 2.23–2.25). This risk decreases over the first year following a cancer diagnosis, but then increases over time [37]. Although a clear cause for the initial decrease in risk has not been established, it may be associated with the removal of the primary tumour and initial treatment resulting in attenuation of the risk from the cancer itself. Additionally, chemotherapy-induced thrombocytopenia may further attenuate this risk [53], as would the use of venous thromboembolism prophylaxis as a routine measure in higher risk patients [54]. For patients younger than 40 years, breast cancers and lymphomas carried the greatest risk of cardiovascular-related late mortality, whereas for those over 40 years, prostate, colorectal, breast, and lung cancers conferred the greatest risk [37]. An analysis of 628 breast cancer survivors supported this finding, demonstrating a hazard ratio of 1.79 (95% CI 1.53–2.09) for CVD risk compared to cancer-free controls [55]. A US study focusing on stroke revealed similar outcomes, with a 2019 analysis of Surveillance, Epidemiology, and End Results data showing a standardized mortality ratio of 2.17 (95% CI 2.15–2.19) in patients with cancer [56]. A third analysis of the same data indicated that CVD was the second most common cause of death amongst US cancer survivors who were more likely to die from CVD than the general population [57]. Ward et al. [58] explored this in greater detail in patients with endometrial cancer, showing that in patients with low-grade cancer, CVD posed the greatest mortality risk, although this may be confounded by the use of hormonal therapy in this cancer type and the fact that patients with low-grade cancer may receive higher doses of therapy given its curative intent. Weberpal et al. [59] in their analysis of breast cancer survivor data from the Surveillance, Epidemiology, and End Results database present a contrasting view to the previously described studies, demonstrating lower heart-related mortality when compared to the general population. The study’s strengths lie in its adjustment for competing risks and large sample size (*n* = 347,476), although it focuses on a specific subgroup of patients and its findings may therefore not be applicable to other cancer types [59]. In addition to not accounting for competing risks, other studies exploring CVD-related mortality in cancer survivors may also lack direct comparison with a cancer-free control group, limiting their ability to accurately capture the extent of the problem [60, 61]. These studies do, however, allow examination of cardiovascular mortality trends and prognostic factors associated with CVD-related death in cancer survivors.

## Prevention, Screening, and Management of Cancer-Related Cardiovascular Disease

Perhaps the most compelling argument for investigating CVD in cancer survivors is the fact that it can be managed and, in some cases, prevented if identified early. Much of the preventative strategies focus on risk factor management (e.g. timely control of dyslipidaemia and treatment-induced diabetes, dietary and exercise interventions, blood pressure control) [49, 62]. Importantly, some anti-cancer therapies, such as radiotherapy [63] and hormone therapies [64], can play a role in the development of the metabolic syndrome and exacerbate pre-existing cardiovascular risk factors. While a balance must be maintained between ensuring adequate tumour control and minimizing adverse effects of treatment, sufficient consideration to mitigation of cardiovascular risk, particularly if the patient is responding well to treatment, is typically lacking. This may not be unreasonable in patients with incurable cancers whose life expectancy is predicted to be short. However, the advent of new, more effective cancer treatments has dramatically increased the life expectancy of patients with cancer, thereby demanding more proactive measures in reducing cardiovascular risk in this cohort.

Table 1 summarizes some of the options for prevention, screening, and management of cancer-related cardiovascular disease discussed below. Note that the evidence supporting each of these measures as effective for use in cancer survivors varies and is discussed further in “Screening and Cardiovascular Risk Stratification”, “Prevention and Management of Cardiovascular Disease”, and “Guidelines Regarding Cancer-Related Cardiovascular Disease Prevention and Management”.

**Table 1:** Strategies for the prevention, screening, and management of cardiovascular disease in cancer survivors

Pre- and intra-treatment screening measures • Cardiac biomarkers: troponin I and T • Natriuretic peptides: B-type NP, N-terminal pro-BNP • Electrocardiogram: QTc prolongation • Echocardiogram: left ventricular ejection fraction, global longitudinal strain Cardiovascular risk stratification^+^ • 2013 ACC/AHA guideline on the assessment of cardiovascular risk: a report of the American College of Cardiology/American Heart Association Task Force on Practice Guidelines • 2019 ACC/AHA guideline on the primary prevention of cardiovascular disease: a report of the American College of Cardiology/American Heart Association Task Force on Clinical Practice Guidelines • National Heart Foundation (Australia): absolute CVD risk clinical guidelines • American Heart Association: Life’s Simple 7 framework Preventative measures • Prehabilitation, including high-intensity interval training • Dietary interventions, including optimization of nutrition and caloric restriction • Risk factor modification through medications including but not limited to anti-hypertensives and statins • Treatment modifications including dose reduction of radiotherapy, concurrent administration of cardioprotective medications with cardiotoxic chemotherapy, modifications in treatment modality and delivery	

^+^ Note that these measures are not specific for patients with cancer and were validated for use in the general population

### Screening and Cardiovascular Risk Stratification

The 2020 European Society for Medical Oncology recommendations for the management of cardiac disease in cancer patients identify a number of timepoints at which screening for cardiac disease and risk factors should occur [65]. Similarly, the American Society of Clinical Oncology Clinical 2017 Practice Guideline on Prevention and Monitoring of Cardiac Dysfunction in Survivors of Adult Cancers provides recommendations for risk stratification and preventative strategies although notably focusing on the impact of treatment on ventricular dysfunction and heart failure, with little mention of atherosclerotic CVD [66]. Risk factor screening pre-treatment undoubtedly forms an important part of management [67, 68]. The European Society for Medical Oncology guidelines also suggests that baseline and intra-treatment measurement of cardiac biomarkers, such as troponin I and T [69, 70] and natriuretic peptides [71], may have some utility in identifying patients at high risk of developing cardiotoxicity from chemotherapy treatment, although the evidence for routine screening of biomarkers is not clear. However, baseline and intra-treatment electrocardiogram screening for QTc prolongation and evaluation of left ventricular ejection fraction, particularly in patients receiving cardiotoxic drugs, is recommended as a routine measure [65]. Global longitudinal strain, an early measure of cardiotoxicity with good diagnostic and prognostic utility, is a newer measure assessed via echocardiogram that can be used in pre- and intra-treatment screening [72]. Notably, there is a lack of post-treatment recommendations for ongoing surveillance of cardiovascular risk in cancer survivors, with most recommendations focusing on pre- and intra-treatment strategies. Several programs, such as Passport for Care, exist for paediatric cancer survivors but such programs are rare in the adult population [73]. Broader guidelines for post-treatment care of cancer survivors generally neglect CVD surveillance, instead of focusing on monitoring for cancer recurrence. Long-term surveillance is particularly important given the ongoing risk of CVD extending well after active treatment.

Risk stratification plays an important role in the prevention and management of CVD in the general population. The American College of Cardiology and the American Heart Association (AHA) has developed both a 2019 guideline on the prevention of CVD [74] and a 2013 guideline for cardiovascular risk assessment [75], recommending the use of the Pooled Cohort Equations for estimation of atherosclerotic cardiovascular disease in the general population. The AHA’s Australian counterpart, the National Heart Foundation of Australia, has a guideline for the management of absolute CVD risk [76] along with a widely used online CVD risk calculator based on the Framingham Risk Eq.  [77] The European Society of Cardiology’s 2021 guideline suggests the use of the SCORE2 (Systematic Coronary Risk Estimate 2) tool for risk stratification in healthy individuals [78]. However, there are no validated risk assessment tools, for survivors of adult cancer [79]. Several organizations have developed consensus statements on risk assessment in specific cancer types and treatment modalities, but there is no broad recognition of the impact of cancer and cancer treatment on CVD risk in survivors of cancer in these statements. Kolominsky et al. [79] suggest that in the absence of such guidelines, the AHA Life’s Simple 7 framework can be used as a tool for CVD risk stratification and counselling in survivors while recognizing that these tools do not account for cancer-related cardiovascular risk, nor the significant impact of specific anti-cancer treatment modalities. Mohammed et al. [80] suggest that such risk calculators severely underestimate cardiovascular risk in cancer survivors, and that these patients should therefore receive aggressive risk factor modification even if their risk score using conventional tools is ‘low’.

### Prevention and Management of Cardiovascular Disease

In addition to screening tools and risk stratification, several interventions that can reduce CVD incidence in cancer survivors also exist and include rehabilitation and exercise interventions, optimization of nutrition, and cardiovascular-specific therapeutics.

The concept of “prehabilitation” is an increasingly popular strategy to mitigate the risk of decline during cancer treatment [8], and provides a safe, low-cost avenue to prevent CVD in survivors. Amongst several types of prehabilitation, high-intensity interval training is associated with improvement of vascular function [81] and peak oxygen consumption [82], the latter being strongly linked to cardiovascular mortality [83]. The recently published ERASE trial demonstrated improved cardiorespiratory fitness following a 12-week high-intensity interval training program in patients with prostate cancer under active surveillance, providing some evidence for a benefit of exercise in those not undergoing treatment [84]. Exercise both during and after cancer treatment can also improve cardiorespiratory fitness and CVD outcomes [85].

Dietary intervention is another avenue being investigated for its efficacy in decreasing CVD incidence. There is increasing evidence supporting the optimization of nutrition for improvement of long-term outcomes and mortality in cancer survivors [8], although there is limited evidence for reduction of cardiovascular risk specifically. Conversely, the Alliance A011401 trial is currently exploring the impact of caloric restriction and physical activity on CVD incidence as a secondary outcome in breast cancer survivors and may provide some insight into the efficacy of dietary interventions [86].

Drug treatments which modify cardiovascular risk factors may also play a role in attenuating CVD risk in cancer survivors. Given the ability of several chemotherapeutic agents, particularly VEGF-inhibitors [87], to induce hypertension, it may be reasonable to employ the use of anti-hypertensives such as dihydropyridine calcium channel blockers [88]; however, there is little evidence supporting the use of one anti-hypertensive agent over another. There is also some evidence supporting the cardioprotective effects of statin treatment in patients treated with anthracyclines [89], although these benefits have not been explored in cohorts not exposed to cardiotoxic chemotherapy. As with ECG screening, these interventions have a pre- and intra-treatment focus, and typically do not extend beyond the treatment period.

It is also worth considering the role of treatment modifications in reducing cardiovascular risk. For example, the administration of doxorubicin in PEGylated liposomal form can reduce anthracycline-mediated cardiotoxicity [90]. Concurrent administration of dexrazoxane during anthracycline treatment may also provide cardioprotection in specific cancer types and age demographics [91]. Of note, the use of remote ischemic conditioning, subjecting tissue remote from the heart to brief, reversible episodes of ischaemia and subsequent reperfusion, is a low-cost novel intervention that has been proposed for use in patients with cancer to prevent anthracycline-mediated toxicity [92]. While this technique is currently only evidenced in animal models [93, 94], the ongoing ERIC-ONC trial [95] and RESILIENCE trial [96] may provide some evidence for the use of remote ischemic conditioning in human patients with cancer receiving anthracycline-based treatment. For those treated with thoracic radiation, the use of advanced radiation techniques such as stereotactic ablative radiotherapy and/or dose reductions may additionally be considered, although Cutter et al. [97] suggest that individual risk assessment is required to determine whether this is appropriate. Similarly, proton therapy has been shown to be effective in reducing the cardiac impact of radiation [98]. Patient-based techniques have also shown benefits for cardiac sparing: these include deep inspiration breath holds [99] and prone positioning [100].

### Guidelines Regarding Cancer-Related Cardiovascular Disease Prevention and Management

Beyond the 2020 European Society for Medical Oncology recommendations for the management of cardiac disease in cancer patients throughout oncological treatment [65] and a 2016 European Society of Cardiology position paper on cardiovascular toxicity [101], there are few evidence-based guidelines that provide recommendations for preventing and managing CVD risk in long-term survivors of cancer. The AHA has recognized the need for cardio-oncology rehabilitation [102] given the intersection between breast cancer and CVD [67], but has not published any specific guidelines. There is some mention of the field of cardio-oncology in the National Comprehensive Cancer Network’s 2018 Survivorship Clinical Practice guidelines, but there is a focus on anthracycline-induced cardiac toxicity [103]. Notably, the newly created Australia and New Zealand Cardio-Oncology Registry will track cardiac dysfunction related to cancer therapy as well as interventions targeting its prevention, although the registry will predominantly focus on chemotherapy-induced cardiotoxicity [104]. Future guidelines should take a more holistic approach, providing recommendations to prevent CVD in all cancer survivors irrespective of whether they have been exposed to known cardiotoxic treatments. The inclusion of recommendations for post-treatment care and ongoing long-term screening would also allow all cancer survivors to receive appropriate cardiac follow-up.

## Challenges Investigating Cardiovascular Risk in Adult Cancer Survivors

There are several variables that can be difficult to assess in the analysis due to data availability. For example, it can be difficult to separate the impact of a cancer itself and cancer treatment. Much of the large-scale research in adult cardio-oncology makes use of national registry–based data to assess CVD incidence and mortality [9–12]. While some of these registries capture limited treatment data, very rarely is specific treatment modality, dosage, and duration data recorded. Without such treatment data, it can be difficult to determine whether CVD risk in cancer survivors is a result of the patient’s cancer, the treatment they have received, or a combination of both. In a similar vein, detailed cancer stage data is often not available in registries or cohort studies. Given the evidence that metastatic disease may confer greater CVD risk than local cancers [9, 24], a lack of staging data can limit analyses. Data on baseline cardiovascular risk, relevant comorbidities (e.g. dyslipidaemia, hypertension, diabetes), and shared risk factors (e.g. smoking, physical inactivity, alcohol consumption, poor nutrition) must also be available to allow for an accurate assessment of the impact of cancer on CVD incidence. Data on mortality events is similarly important to adjust for death as a competing risk.

We must also consider that as cancer treatment evolves, the CVD risk posed by treatments changes. Some modalities, such as radiotherapy, have conferred less risk over time. Mulrooney et al. [105] report that modifications in modern radiation protocols resulted in a reduced risk of coronary artery disease from the 1970s to the 1990s. Although this study was conducted in adult survivors of childhood cancers, it nonetheless illustrates that treatment-induced CVD risk can improve over time. In adult patients with breast cancer, the mean cardiac radiation dose has decreased from 13.3 Gy in the 1970s to 2.3 Gy in 2006 [106]. Conversely, newer chemotherapeutic classes such as certain targeted therapies [107] have been found to pose increased CVD risk when compared to some older agents. Without detailed cancer treatment data, it is difficult for studies to account for the varying impact of different treatment modalities and protocols on CVD risk and incidence.

The requirement for long-term follow-up to accurately assess CVD risk also makes cardio-oncology research difficult. As previously described, coronary heart disease risk can remain elevated even 10 years following a cancer diagnosis [10]. Similarly, stroke risk is highest during the first 6 months post-cancer diagnosis but remains elevated for 10 years post-diagnosis [9]. The risk of some events, such as heart failure and cardiomyopathy, can even continue to increase over 12 + years [11]. Retrospective analysis of cancer registries can provide meaningful data with minimal logistical burden, facilitating large sample sizes and long follow-up time. The alternative, prospective studies, provide a greater level of evidence but require considerable funding to maintain follow-up over a decade-long period.

The predominance of paediatric cohorts in cardio-oncology research should also be noted. The Childhood Cancer Survivor Study (CCSS) provided valuable data in establishing a link between childhood cancer and CVD. Mulrooney et al. [108] found that childhood cancer survivors from the CCSS were more likely than their cancer-free siblings to experience congestive heart failure (hazard ratio [HR] 5.9, 95% CI 3.4–9.6), myocardial infarction (HR 5.0, 95% CI 2.3–10.4), pericardial disease (HR 6.3, 95% CI 3.3–11.9), and valvular disease (HR 4.8, 95% CI 3.0–7.6), with anthracyclines and cardiac radiation conferring the greatest risk. The CCSS, in combination with several other studies which support the increased CVD risk in childhood cancer survivors [109], laid the groundwork for evidence-based CVD risk prediction models in childhood cancer survivors [110] and guidelines for long-term follow-up in this cohort [111]. The fact that adult cardio-oncology is a much newer field has meant that it is some way behind its paediatric equivalent in regard to a comprehensive literature base and the presence of evidence-based guidelines.

Table 2 summarizes the challenges in investigating cardiovascular risk discussed in this section.

**Table 2:** Challenges in investigating cardiovascular risk in cancer survivors

• Separating the impact of cancer treatment from the impact of the cancer itself • Disentangling the impact of cancer and cancer treatment from that of shared risk factors (e.g. smoking, diet, physical activity, comorbidities) and normal ageing • Adjusting for cancer-related factors in analysis that are often not available in databases (e.g. cancer treatment, cancer stage) • Adjusting for non-cancer-related factors in analysis (e.g. shared risk factors, comorbidities, competing risk of death) • Adjusting for the changing cardiovascular risk posed by cancer treatments as these treatments evolve over time • Ensuring appropriate follow-up to capture late cardiovascular effects of treatment • Capturing data in adult cohorts in a field where much of the data is in paediatric cohorts	

## Future Directions

Cardio-oncology is a rapidly evolving field and is supported by several large-scale studies confirming the impact of cancer on subsequent CVD. However, more studies in adult cohorts with extensive follow-up periods are required to inform evidence-based guidelines on recommendations for post-treatment screening and management in adult cancer survivors. Although an abundance of research exists exploring long-term CVD outcomes in childhood cancer survivors, the majority of diagnoses are hematological in nature, with less than half of all diagnosed cancers in the CCSS being solid tumours [112], limiting the generalizability of this data to an adult survivor population that suffers from vastly different types of cancers with different underlying mechanisms and different anti-cancer therapies.

Future research investigating the link between CVD and cancer in adult cancer survivors must aim to account for shared risk factors, different treatment modalities, doses, and durations, the variation in risk across both cancer types and stages, and competing risks. In doing so, factors that increase susceptibility to the cardiotoxic effects of cancer and cancer treatment can be identified and subsequently used to identify high-risk patients. The literature must also be updated as new treatment protocols and modalities are introduced, given that each type of treatment carries with it a different CVD risk.

Randomized-controlled trials investigating CVD screening tools and interventions in adult cancer survivor cohorts are also vital, although this can be difficult given the need for long-term follow-up in capturing CVD risk. Without this research, it is difficult to determine how best to screen for cardiovascular disease in cancer survivors, as well as when to intervene prophylactically. Furthermore, while pre- and intra-treatment care is important, there is undoubtedly a gap in the post-treatment space. The utility in post-treatment care lies in the opportunity to reduce comorbidity burden, improve quality of life, and reduce cardiovascular mortality. The efficacy of common CVD risk factor modification treatments (e.g. statins, anti-hypertensives) in adult cancer survivor cohorts should also be validated.

Ultimately, the greatest gap in cardio-oncology is the lack of evidence-based guidelines for long-term CVD prevention and management in cancer survivors. For clinicians to incorporate routine surveillance for, and preventative management of, CVD in the care of cancer survivors, evidence-based recommendations need to be generated by national and international oncology and cardiology organizations. In addition to guidelines, adequate training must be provided to cancer specialists and primary care physicians to ensure that they are well-informed about the CVD risks in cancer survivors. This should extend beyond pre-treatment screening and intra-treatment surveillance for treatments known to be cardiotoxic and recognize the long-term risk of CVD posed by all cancer types and treatment modalities. A multi-disciplinary approach to cardio-oncology is also vital, requiring input from not only cancer specialists but also other healthcare professionals including primary care physicians, cardiologists, and allied health professionals, in order to provide appropriate, specialized cardiovascular care to cancer survivors.

Table 3 summarizes some of the options for future research and changes to clinical practice for cardio-oncology.

**Table 3:** Future directions for cardio-oncology in research and clinical practice

Opportunities for research: cardiovascular disease (CVD) incidence and driving mechanisms • Investigate CVD risk in adult cancer survivor cohorts with adjustment for shared risk factors, cancer-related variables, anti-cancer treatment, and competing risks • Explore long-term risk of CVD in cancer survivors using longitudinal follow-up • Explore cancer-related mechanisms driving CVD in cancer survivors, both direct cancer- and treatment-related Opportunities for research: screening and interventions • Investigate utility of routine pre- and intra-treatment CVD screening strategies for CVD in cancer survivors • Conduct randomized-controlled trials investigating the role of prevention and management of CVD during active treatment on CVD in cancer survivors • Investigate post-treatment CVD surveillance programs for adult cancer survivors • Investigate strategies for active intervention in cancer survivors through pragmatic clinical trials Opportunities for clinical practice • Publish guidelines for screening, prevention, and management of CVD in cancer survivors both pre- and post-treatment • Train healthcare professionals in managing CVD risk in cancer survivors, along with educating patients about their individual risk profile • Encourage a multi-disciplinary approach to cancer survivorship care with input from medical specialists, primary care providers, and allied health professionals • Include CVD screening in routine long-term cancer survivor surveillance programs • Implement evidence-based interventions (e.g. dietary and exercise programs, risk factor management) to mitigate CVD risk in cancer survivors	

## Conclusion

Cancer and cancer treatment can accelerate aging, of which CVD is a common sequela. There is evidence supporting a link between cancer and increased risk of cardiovascular disease, which can remain elevated for up to a decade post-diagnosis, along with reductions in quality of life and increases in mortality. Some strategies exist to mitigate this risk and treat CVD in cancer survivors but given the relative youth of the field of cardio-oncology, there are few evidence-based recommendations from professional organizations surrounding the management of CVD in this cohort. Evidence-based clinical guidelines for the management of cancer-associated cardiovascular disease and a multi-disciplinary effort in cardio-oncology research, education, and clinical care are required to improve cardiovascular outcomes for cancer survivors.

## Supplementary Information

Below is the link to the electronic supplementary material.

Supplementary file1 (DOCX 16 KB)
