# Supplementary material for: Cardiovascular Disease in Adult Cancer Survivors: a Review of Current Evidence, Strategies for Prevention and Management, and Future Directions for Cardio-oncology
Source: Curr Oncol Rep. 2022 Jul 7;24(11):1579–92. doi: 10.1007/s11912-022-01309-w (PMC9606033; doi:10.1007/s11912-022-01309-w)
Supplement: Supplementary file 1 — Supplementary file1 (DOCX 16 KB) [file 11912_2022_1309_MOESM1_ESM.docx]

**Supplementary Material**

**Literature search strategy**

**Databases searched:** Google Scholar, PubMed, OVID Medline

**Search strategy:**

An initial scoping literature search was conducted using Google Scholar and PubMed using the search terms cancer; cardiovascular disease; stroke. This search was used to gauge the availability of literature and identify landmark studies investigating the link between cancer and cardiovascular disease.

A more comprehensive search was conducted using OVID Medline (21 September 2021) focussing on recent, English language studies. An additional search was conducted on stroke and cerebrovascular disease due to the abundance of studies only focussing on this aspect of cardiovascular disease.

| *Search 1* | *Search 2* |
| --- | --- |
| Title: Mesh search for cancer AND | Title: Mesh search for cancer AND |
| Title: String search for cardiovascular disease OR heart disease OR cardiovascular disease related terms | Title: String search for stroke OR cerebrovascular disease OR stroke related terms |
| Filter: English language | Filter: English language |
| Filter: 2018-Current | Filter: 2018-Current |

Search 1 yielded 264 articles while search 2 yielded 115 articles, totalling 379 articles.

**Additional screening:**

Articles were title screened and included on the basis of being relevant to one of the following topics:

- The link between cancer/cancer treatment and the incidence of cardiovascular disease
- Strategies to prevent or manage cardiovascular disease in cancer survivors
- Cardiovascular mortality in cancer survivors

A total of 120 articles remained after title screening. All articles were sorted into three groups: link between cancer and cardiovascular disease, prevention and management of cardiovascular disease, and cardiovascular disease mortality.

Articles were subsequently abstract screened, with high quality articles being selected for inclusion in this review. Features used to identify high quality articles included large sample size, presence of a control group, separation and adjustment for treatment factors, separation of specific types of cardiovascular disease, long follow up time, and multi-site or national/international recruitment.
